# Genital Lymphedema and How to Deal with It: Pearls and Pitfalls from over 38 Years of Experience with Unusual Lymphatic System Impairment

**DOI:** 10.3390/medicina57111175

**Published:** 2021-10-28

**Authors:** Juste Kaciulyte, Leonardo Garutti, Davide Spadoni, Jonathan Velazquez-Mujica, Luigi Losco, Pedro Ciudad, Marco Marcasciano, Federico Lo Torto, Donato Casella, Diego Ribuffo, Hung-Chi Chen

**Affiliations:** 1 Department of Plastic and Reconstructive Surgery, China Medical University Hospital, China Medical University, Taichung 40447, Taiwan; justekc@gmail.com (J.K.); leonardo.garutti.93@gmail.com (L.G.); davidespadoni03@gmail.com (D.S.); Drjonathan.vm@gmail.com (J.V.-M.); luigi.losco@gmail.com (L.L.); pciudad@hotmail.com (P.C.); dott.marcomarcasciano@gmail.com (M.M.); federicolotorto@gmail.com (F.L.T.); 2 Unit of Plastic and Reconstructive Surgery, Department of Surgery “P. Valdoni”, Policlinico Umberto I, Sapienza University of Rome, 00185 Roma, Italy; diego.ribuffo@uniroma1.it; 3 Department of Biotechnology and Life Sciences, Division of Plastic and Reconstructive Surgery, University of Insubria, 21100 Varese, Italy; 4 Odontostomatological Science and Maxillo-Facial Surgery Department, Policlinico Umberto I, Sapienza University of Rome, 00185 Roma, Italy; 5 Department of Translational Research and New Technologies in Medicine and Surgery, University of Pisa, 56126 Pisa, Italy; 6 Department of Plastic, Reconstructive and Burn Surgery, Arzobispo Loayza National Hospital, Lima 15082, Peru; 7 UOC Chirurgia Oncologica Della Mammella, Department of Medicine, Surgery and Neurosciences, University Hospital of Siena, 53100 Siena, Italy; donatocasella@gmail.com

**Keywords:** genital lymphedema, rare lymphedema, debulking surgery, physiologic surgery, multimodal approach, targeted therapy

## Abstract

*Background and Objectives*: Conservative treatment represents an essential pillar of lymphedema management, along with debulking and physiologic surgeries. Despite the consistent number of treatment options, there is currently no agreement on their indications and possible combinations. When dealing with unusual lymphedema presentation as in the genitalia (Genital Lymphedema—GL), treatment choice becomes even more difficult. The authors aimed to present their targeted algorithm of single and combined treatment modalities for rare GL in order to face this paucity of information. *Materials and Methods*: Data were collected from a prospectively maintained database since January 1983, and cases of GL that were managed in the authors’ department were selected. Only patients that were treated in the authors’ institution and presented a minimum follow-up of 3 months were admitted to the current study. *Results*: From January 1983 to July 2021, 19 patients with GL were recruited. All the patients were male, and their ages ranged from 21 to 73 years old (average: 52). Ten cases (52.6%) presented with ISL (International Society of Lymphology) stage I, five (26.3%) were stage II and four (21.1%) were stage III. GL was managed with conservative treatment (12 cases), LVA (LymphaticoVenous Anastomosis) (3) or surgical excision (4). In a mean follow-up of 7.5 years (range: 3 months—11 years), no major complications occurred, and all cases reached improvements in functional and quality of life terms. *Conclusions*: Contrary to the predominant thought of the necessity to avoid surgery in unusual lymphedema presentations such as GL, they can be managed using targeted multimodal approaches or by adapting well-known procedures in unusual ways to achieve control of disease progression and improve patients’ quality of life.

## 1. Introduction

The term “lymphedema” defines a chronic and progressive pathologic accumulation of protein-rich lymphatic fluid in the interstitial space due to lymphatic vessels and/or lymph nodes impairment [1]. Lymphedema onset is insidious, starting with slight tightness of clothing and a feeling of heaviness and fatigue in the affected area. Over time, edema becomes visible and develops from pitting to non-pitting and to soft tissue proliferation and fibrosis that impairs, even more, the already jeopardized function of the lymphatic system [2]. Overall, 140–250 million people are estimated to be affected by lymphedema worldwide, and lymphedema secondary to Wuscheria banrofti infection represents the most common form, affecting more than 90 million people [3].

Regardless of the etiology, the primary goal of lymphedema management consists of symptom control and reducing the progression of the disease. Conservative treatment represents the first-line approach, in particular the Complete Decongestive Therapy (CDT) that consists of manual manipulation, compression bandaging, exercise at home and skincare [4]. While CDT continues to be an essential pillar of any treatment plan, several surgical procedures were developed to reduce pathologic adipose and fibrotic tissue excess and to stop lymphedema progression. The debulking surgeries aim to remove the diseased skin and subcutaneous tissue that present irreversible changes. Suction Assisted Lipectomy (SAL) is commonly performed for initial soft tissue accumulation, while in cases of severe fibrosis, direct excision becomes necessary with more invasive procedures as Radical Reduction with Preservation of Perforators (RRPP) or modified Charles’ procedure [5,6]. On the other hand, recent advances in microsurgery and understanding of lymphedema pathophysiology introduced novel physiologic procedures that aim to restore the impaired lymphatic flow, such as Lymphaticovenular Anastomosis (LVA) [7,8,9] and Vascularized Lymph Node Transfer (VLNT) [10]. However, despite the consistent number of surgical treatment options, there is currently no agreement on indications, timing and possible combination of these procedures [11].

Lymphedema management becomes even more difficult and obscure when dealing with unusual presentations, such as genital lymphedema (GL). The involvement of external genitalia represents an uncommon form, accounting for 0.6% of lymphedema cases worldwide [12]. Lymphatic obstruction secondary to filariasis is the most common cause, but other clinical conditions such as cancer therapies, hidradenitis suppurative, primary presentations or heart, liver and kidney dysfunctions are more common causes in the developed countries. Less frequently, GL occurs after exogenous substances injections, such as paraffin or silicone [13]. Recurrent infections and limitations in daily movements and activities, hygiene procedures and social and sexual life make the GL an extremely uncomfortable condition that severely impairs the patient’s quality of life [14]. Due to the rarity of the condition, diagnostic criteria and modality and timing of therapies have been debated without reaching a consensus to date [15].

By taking into account the paucity of information in the literature on GL and other unusual lymphedema presentations, such as the head and neck lymphedema, chylous ascites or primary lymphedema associated with vascular malformations, the authors of the current paper aimed to show their results in managing these rare diseases, particularly in dealing with GL. Thanks to over 38 years of experience, the pearls and pitfalls of single and combined treatment modalities are described, with the goal to present the authors’ treatment algorithm targeted for each case.

## 2. Materials and Methods

This is an institutional board-approved review of a prospectively maintained database performed at China Medical University Hospital in Taichung, Taiwan. The retrospective analysis was conducted after review and approval by the Research Ethics Committee (CMUH106-REC1-111[CR1]), and the Helsinki declarations were strictly adhered to in the course of this study. Informed consent was taken from all the patients included in the study and for publishing diagnostic studies and clinical images.

The database presented data since January 1983, and the current retrospective selection was carried out, including cases of unusual lymphedema presentations that were admitted and treated in the Plastic and Reconstructive Surgery Department of the China Medical University Hospital in Taichung, Taiwan. The main inclusion criterion was the presence of lymphedema in the genitalia. Only patients that were treated in our institution and presented a minimum follow-up of 3 months were admitted to the current study. Patients that failed to present the minimum follow-up or were subsequently treated in other facilities were excluded from the study.

**Figure 1:**
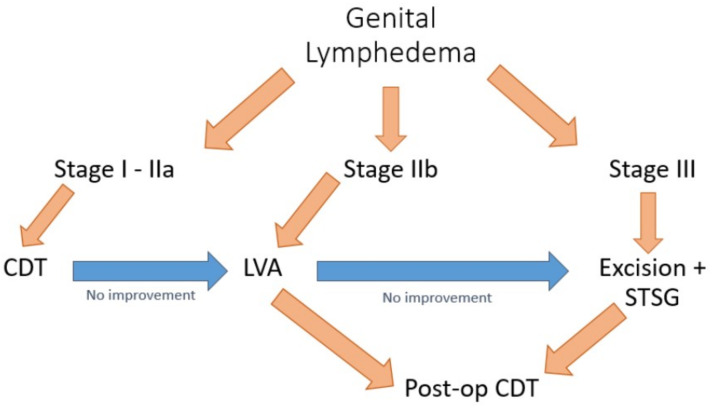
Treatment algorithm applied to manage GL.

At the moment of admission, patients with GL were staged according to the International Society of Lymphology (ISL) system. Figure 1 shows the treatment algorithm applied in our unit.

Stage I and mild presentations of Stage II were approached with conservative therapy first, consisting of compression. In cases of conservative management failure after a minimum of 3 months, physiologic surgery with LVA was proposed to Stages I and II. For cases of chronic and progressed Stage III GL, instead, excisional procedures represented the solution in order to reduce fibrotic tissue accumulation and reduce deformity and functional impairments. The debulking procedure was carried out with circumcision by removing all the affected skin and subcutaneous tissues deep to the fascia, carefully preserving testes and spermatic cords. The scrotal septum was also preserved to achieve a favorable appearance, similar to the tunica vaginalis that was identified and inverted. Moreover, when feasible, patent subcutaneous lymphatic vessels were visualized with injections of patent blue and preserved. Before closure, testicles and spermatic cords were sutured together with absorbable stitches in order to prevent the formation of bifid scrotum. Local skin flaps allowed coverage of scrotum structures, taking care to perform a midline suture to the retained scrotal septum in order to reconstruct the scrotal raphe. When complete coverage with flaps was not possible, meshed split-thickness skin grafts (STSG) allowed resembling scrotal rugae, and positional gravity progressively expanded the graft, giving it a natural pendulous appearance. When the penis was involved, attempts to preserve dartos were made during excision in order to enhance the subsequent extensibility of the graft and mobility. Skin excision was carried out until the coronal sulcus or to the glans, when affected, to reduce the risk of recurrence due to any residual tissue. In contrast with the scrotum area, coverage with unmeshed STSG was recommended for sexually active patients in particular. The graft was secured at the penis base and the neo-raphe, with tucked-in sutures and tied-over silk sutures that minimize shearing forces and improve the graft’s take.

Post-operative CDT was prescribed to all patients for at least 6 months in order to maintain results and prevent recurrences.

Outcomes were assessed in terms of edema reduction and ISL stage regression, skin quality and functional reported outcomes.

## 3. Results

**Figure 2:**
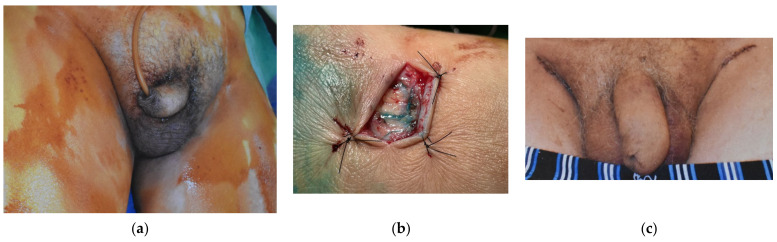
A male patient, 56 years old, presented to our unit with GL stage II involving the scrotal area, subsequent to prostatic cancer treated with prostatectomy, radiation and chemotherapy (**a**) The scrotal lymphedema did not respond to 6 months of CDT, so it was treated with LVA procedure: (**b**) during surgery, ICG was infused near the groin area and the lymphatic ducts were mapped with an ICG camera. Moreover, patent blue was injected to visualize the lymphatic vessels directly. One LVA was performed on each side of the groin area. Three months after surgery (**c**), there was remarkable improvement of swelling at the genital area, and the patient felt more comfortable, with easier passage of urine reported.

**Figure 3:**
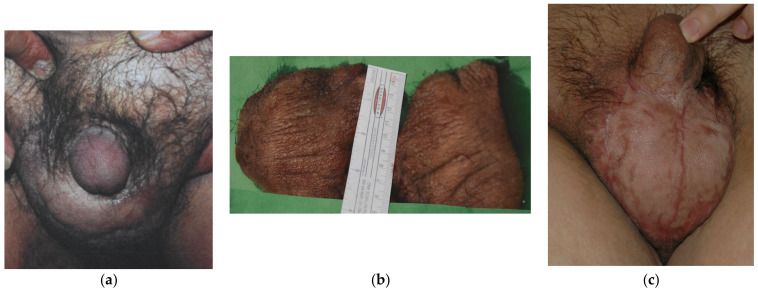
A male patient, 45 years old, presented to our unit with GL stage III involving the scrotal area after radiation for retroperitoneal malignancy. The scrotal lymphedema was almost completely hiding the penis. (**a**) Excisional surgery and scrotum reconstruction with meshed STSG were performed: the picture (**b**) shows indurated skin and subcutaneous tissue due to chronic inflammation that were excised. One year after surgery, there was no more inflammation and discharge in the genital area. The urine passage was smooth (**c**).

Since 1983, a total of 19 cases of GL have been managed until July 2021 in our unit. All the patients were male, and their ages ranged from 21 to 73 years old (mean age 52 years old). Seventeen (89.5%) cases of GL were cancer-related, subsequent to pelvic cancer treatment, while two (10.5%) cases were consequent to chronic inguinal hidradenitis suppurative. Ten cases (52.6%) presented with ISL stage I, five (26.3%) were stage II and four (21.1%) were assessed as stage III. Twelve (63.2%) patients presented mild symptoms with stage I GL (10 cases) or could not undergo surgical treatment due to persistent malignancy (two cases of GL stage II), and they were managed with conservative treatment solely. The other seven patients failed to improve after 3 months of conservative approach and underwent LVA procedure (3 stage II patients; 15.8%) (Figure 2) or needed for surgical excision of the scrotum and penis fibrotic tissues and subsequent coverage with STSG (4 stage III cases; 21.1%) (Figure 3).

The average follow-up was 7.5 years (range: 3 months–11 years), and all patients showed consistent edema reduction and soft tissue quality improvement in terms of self-perceived swelling and elasticity. Only one case (5.3%) treated with LVA did not achieve complete edema resolution but improved from stage II to stage I GL. The other 18 patients (94.7%) experienced complete resolution of GL, with no relapse during follow-up. Patients that were treated with surgical excision reported difficulties in urinating prior to treatment, with the necessity to compress the swollen penis before urine could pass. After surgery, the urine passage was smooth with no necessity for compression.

## 4. Discussion

Usually, lymphedema involves upper or lower limbs, and Wuscheria banrofti infection represents the most common cause worldwide [3]. Filariasis is the most frequent cause even of GL, but the involvement of external genitalia represents an uncommon lymphedema presentation, accounting for 0.6% of lymphedema cases worldwide [12]. Due to the rarity of GL, diagnostic criteria and modality and timing of therapies were debated without reaching a consensus to date [15]. Conservative CDT therapy is considered the mainstay treatment for the common limb lymphedema, but its availability is limited for GL due to the shape and location of genitalia. Compression may maintain and even reduce the genital size, but the patient’s inconvenience must be taken into account [16]. Given the poor efficacy of conservative therapy, surgery represents a valid option, especially in cases of excessive enlargement, disfigurement and functional impairment [17]. A variety of physiological and excisional surgical procedures, with or without overlying skin preservation, is offered in the literature, but no consensus on their use has been reached to date [18,19,20,21]. Among the debulking surgeries for GL, the Charles procedure is considered to be the earliest and most radical one, and it was described for the scrotum or for both scrotum and penis [22,23]. As an alternative, surgical excision may be carried out as simple hydrocelectomy, consisting of tunica eversion or excision, Lord’s procedures or as scrotal and penile resection and reconstruction [18]. After the debulking procedure, full-thickness skin grafts (FTSG) [16] or STSG [24] were described for coverage, as well as local skin flaps [25,26,27,28]. Few studies reported any reconstruction after pathologic tissue excision [29,30,31]. For what concerns physiologic procedures to GL treatment, LVA was described by Mukenge et al., in two reports, where the anastomosis was carried out between the lymphatic vessels of the spermatic funiculus and branches of the spermatic veins [32,33].

Physiologic and excisional procedures have been used singularly or in various combinations for more than three decades in our unit. Thanks to the experience gained by the senior author (H.C.C.) in extremity lymphedema treatment [34,35], the same surgeries were adapted to manage unusual lymphedema presentations by changing the area of their application (LVA in groin area for GL or in the face for the head and neck lymphedema), combining them together in targeted protocols for GL or in the presence of associated vascular malformations or redesigning the VLNT concept into the lymphatic cable flap transfer for chylous ascites treatment.

The treatment protocol designed for GL, in particular, consisted in matching the beneficial effects of pre- and post-operative CDT with the low-invasiveness and efficacy of LVA for early GL stages and with a more radical debulking approach and reconstruction with STSG for advanced GL cases.

Contrary to the predominant thought of the necessity to avoid surgery in unusual lymphedema presentations such as GL, our complex targeted protocol aimed to control the disease by addressing its whole aspects. Encouraging results were reached, as all patients improved in function and appearance, and no relapse was registered. Still, surgeons that manage GL and other unusual lymphedema presentations have to keep in mind that they are facing complex cases, for which a wide knowledge of conservative and surgical procedures is necessary. It is mandatory to perform a thorough pre-surgical assessment and to address each particular lymphedema case with the most suitable treatment of all the armamentarium at disposal precisely.

## 5. Conclusions

With the growing knowledge and technology, the uncommon forms of lymphedema can be treated using targeted multimodal approaches or by adapting well-known procedures in unusual ways to provide reasonable control of disease progression and improve patients’ quality of life. More patients with longer follow-up will be necessary to reach consistent results and give more support to the treatment protocol described here.

## Data Availability

The data presented in this study are available on request from the corresponding author. The data are not publicly available due to patients’ privacy issues.
